# Books Reviewed

**Published:** 1868-06

**Authors:** 


### Books Reviewed.

Stillé's Therapeutics and Materia Medica. Third edition, revised and enlarged, in two volumes. Philadelphia: Henry C. Lea, 1868.

Stillé's Therapeutics and Materia Medica requires no general description from us; its character and standing are too well understood by all intelligent physicians to admit of it. The simple announcement of the appearance of a third edition will at once suggest the inquiry, what new subjects have been added? About one hundred pages of new matter have been added, mainly upon the subjects of: *Chromic Acid; Permanganate of Potassa; the Sulphites of Soda, etc.; Carbolic Acid; Nitrous Oxide: Rigoline, and Calabar Bean.* There has also been added an article upon *Bromine*, and that on *Electricity* has been enlarged by an account of recent improvements in apparatus, and the application of this agent to the cure of diseases.

Speaking of the Sulphite of Soda, one of the new substances treated, he says: "Although the power possessed by the sulphites of controlling fermentation had long been known and usefully employed, it was not generally recognized or applied in medicine until attention was attracted to the subject by Dr. Polli of Milan.

Assuming as correct the hypothesis that *in all* contagious and infectious diseases their phenomena are due to a process of zimosis, or fermentation, and recognizing the power of sulphurous acid to cheek fermentation and putrefaction, he concluded that this acid or its salts, must be capable of curing radically the diseases in question. By experiment he found that the fatal symptoms occasioned by the introduction of putrid substances into the veins of animals were modified, suspended, or neutralized by the injection of solutions of the sulphites into the blood. It was also demonstrated that gangrenous and fetid suppurating surfaces, and all foul-smelling discharges were speedily rendered inodorous by the application of these salts in solution. But the clinical proof of their efficacy in disease, which would have gone far to justify the hypothesis of Polii, was not adduced by him, nor has it been conclusively adduced by later observers."

The author now proceeds to relate what a great number of physicians have published of the effects of these salts in *erysipelas*, in *intermittent fever*, *typhoid fever*, *yellow fever*, *purulent infection* or *septicæmia*, and other similar diseases, but finally expresses incredulity in regard to the efficacy of a remedy which has hardly been sufficiently tested. The good sense and discrimination of the anthor is finely illustrated in this newly added chapter, as indeed is shown everywhere in the pages of the book. We regret that we cannot follow up the newly added pages and speak in detail of their contents, but must close, by assuring our readers, that the subjects are treated in a most masterly manner. Stillé's Therapeutics and Materia Medica stands unrivaled in our language; it furnishes all the positive evidence for the materials of medicine required. The negative results of medication have never yet been collected; immortal honor to the author who shall collect and publish the opposite side. The arrangement and general plan of the work before us has contributed to its popularity. The happy combination of therapeutics with materia medica adds greatly to its value, and has established its reputation. It may very truly be regarded as a fountain of all knowledge in its department, and as presented in its third edition embodies what is known in therapeutics and materia medica.

The work of the publisher must not be omitted in this notice, when so many of our new books in medicine are bound in inferior manner. This work comes to us in two volumes, beautifully bound in leather of most enduring and permanent quality, while the typographical execution is unsurpassed. It is truly in all respects a model for imitation and admiration.

---

Chronic Diseases of the Larynx, with a special reference to Laryngoscopic Diagnosis and Therapeutics. By Dr. Adelbert Tobold. Translated from the German and edited by George M. Beard, A. M., M. D. New York: Wm. Wood & Co,, 1868.

Until quite recently the diagnosis of all diseases of the larynx rested upon the greatest uncertainty, arising from the unsatisfactory manner in which the pathological changes occurring in this organ could be observed; since the introduction, however, of the laryngoscope, the diagnosis of this class of diseases has attained to a degree of positiveness and certainty equal to that attained by the opthalmoscope in the diseases of the eye. The development of this science has been so rapid that our text-books treat this important agent (the laryngoscope) in the "*local diagnosis*" of laryngial affections in the most cursory manner, rendering a work which would fully treat this class of diseases an absolute necessity. This necessity appears to us to be fully supplied by the work of Dr. Tobold, and with the extensive and valuable additions by the editor to fully meet all requirements.

The first eleven chapters of the work have been devoted to a general consideration of laryngoscopy; the description and mode of application of the apparatus; the manner of conducting an examination; the laryngial image, and a chapter on rhinitis by the editor. The apparatus described is one of the author's own invention. in which a powerful artificial light for the purpose of illumination, replaces the solar, which latter is regarded as impracticable and unreliable on account of its variableness, the great desideratim in laryngoscopy being a powerful and constant light.

The second part of the work is devoted to a consideration of the chronic diseases of the larynx, and it is here that we would wish to present some of the views of the author upon the interesting subject of tuberculosis laryngitis. The teachings of Trousseau and Belloe have been that phthisis laryngealsis may be of spontaneous development. The author's observations are decidedly opposed to this statement, he regarding "*tuberculosis laryngitis only as the effect of general tuberculosis;*" and furthermore, he says: "*I hold that tuberculosis of the larynx as exclusively a product of tuberculosis of the lung.*" The theory that the breaking up of the cavernous secretion should favor the formation of laryngeal tuberculosis, according to the view of Louis is also opposed by the author, since he says "there are very numerous cases where an affection of the larynx has already far advanced before cavities have been formed in the lungs. Just as little could tuberculous bronchial sputa through contact with yet healthy laryngeal mem brane excite analogous processes unless ulceration of any other kind already existing in the larynx, favors the absorption of tuberculous matter."

We should be happy to place further extracts upon this aud other subjects before our readers, would space permit our so doing. The work highly commends itself to the attention of physicians, since it is the only one in our language fully discussing diseases of the larynx, diagnosed and treated by means of the laryngoscope.

